# Two subsets of regulatory CD8^+^ T cells with differential transcriptome revealed by single cell analysis

**DOI:** 10.1016/j.isci.2025.113512

**Published:** 2025-09-08

**Authors:** Céline Sérazin, Lisa Dugast, Léa Flippe, Mathias Streitz, Désirée-Jacqueline Wendering, Stephan Schlickeiser, Frederik Heinrich, Pawel Durek, Gabriela Maria Guerra, Katrin Lehmann, Mir-Farzin Mashreghi, Harald Wajant, Hans Dieter Volk, Ignacio Anegon, Laurent David, Séverine Bézie, Carole Guillonneau

**Affiliations:** 1 Nantes Université, INSERM, Center for Research in Transplantation and Translational Immunology, UMR 1064, CNRS, 44000 Nantes, France; 2 BIH Center for Regenerative Therapies (BCRT), Charité - Universitätsmedizin Berlin, 13353 Berlin, Germany; 3 Institute of Medical Immunology, Charité - Universitätsmedizin Berlin, 13353 Berlin, Germany; 4 Deutsches Rheuma-Forschungszentrum, Ein Institut der Leibniz-Gemeinschaft, 10117 Berlin, Germany; 5 Division of Molecular Internal Medicine, Department of Internal Medicine II, University Hospital Würzburg, 97080 Würzburg, Germany

**Keywords:** Immunology, Transcriptomics

## Abstract

Although CD8^+^ regulatory T cells (Tregs) were described in the 1970’s, they remain poorly defined compared to CD4^+^ Tregs. Their phenotypic heterogeneity and lack of consensus markers have hindered mechanistic studies and slowed clinical development despite their therapeutic potential. In this study, we performed single-cell RNA sequencing coupled with CITE-seq and TCR-seq on peripheral blood CD8^+^ T cells from four healthy donors, including the CD45RC marker to distinguish pro-inflammatory from pro-regulatory subsets. We analyzed ∼7000 freshly isolated, non-stimulated CD8^+^ T cells and identified two distincts CD8^+^ Tregs subsets, defined by HELIOS or TNFR2 expression, with unique transcriptional and surface marker profiles. Functional assays revealed potent suppressive capacity of the TNFR2^+^CD29^low^CD45RC^low/-^ subset. These findings were independently validated using a publicly available single-cell dataset from four additional individuals. This work provides the most comprehensive profiling of human CD8^+^ Tregs to date and supports their translation to clinical application.

## Highlights

• Single-cell RNA-seq and CITE-seq identify two distinct subsets of human CD8^+^ Tregs
• HELIOS^+^ and TNFR2^+^ CD8^+^ Tregs display divergent transcriptomic and TCR profiles
• TNFR2^+^CD29^low^ CD8^+^ Tregs exhibit potent suppressive function *in vitro*
• Findings are validated in an independent public dataset of 60,000 CD8^+^ T cells

## Graphical abstract

**Figure:**
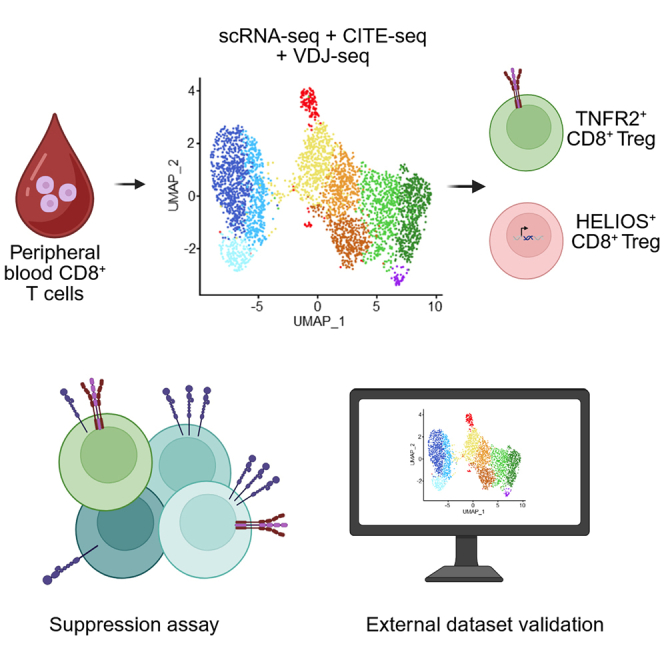

## Introduction

Tregs are key components of the immune system involved in the maintenance of tolerance and acting to suppress immune responses in many pathological situations. The discovery in 1995 of CD25 as a cell membrane marker for their identification correlating with the discovery of a Treg-specific transcription factor forkhead box protein P3 (FOXP3) has been a trigger in the characterization of CD4^+^ Tregs and for their subsequent development for a clinical application.^1^ Since then, numerous efforts have been done for the comprehension of these cells critical in the regulation of the balance of the immune system such as the description of their transcriptional landscape in various compartments and in diseases.^2^^,^^3^ At the opposite, few efforts have been done for a better understanding of CD8^+^ Tregs (or CD8^+^ T suppressor cells as they were originally called in 1972^4^) despite having been described by many groups, but with different phenotype or at least there has been no attempt to correlate one phenotype with another, in multiple pathophysiological situations in different species including humans.^5^^,^^6^^,^^7^ The role of FOXP3 or HELIOS in CD8^+^ T cells has been suggested in human and rodent but poorly described due to their scarcity and subsequent difficulty to study,^8^^,^^9^ thus the existence of a CD8^+^ Tregs lineage-specific transcription factor is still debatable, which together with the absence of consensual and specific surface markers, led to the general disregard of CD8^+^ Tregs for many years.^10^^,^^11^^,^^12^

The rapid development in the last years of technologies and methods to gain detailed information on the transcriptome and proteome at a single cell level has allowed the discovery of rare cell populations, as well as their lineage specifications. However, to date, there has been no attempt to reveal CD8^+^ Treg subsets in blood from healthy donors and understand the heterogeneity within them and relationship between CD8^+^ T cell subsets. Indeed, as for other cell populations, one could expect that CD8^+^ Tregs exist in different identities related to distinct functional characteristics and differentiation pathways and that would fit with the many phenotypes described for CD8^+^ Tregs. So far, our team^13^^,^^14^^,^^15^^,^^16^^,^^17^ and others^18^^,^^19^^,^^20^ have demonstrated that CD8^+^ Tregs identified by low and/or negative expression of the marker CD45RC, one of the isoforms of the CD45 molecule, show potent suppressive activity *in vitro* and *in vivo*, while mouse, rat, and human cells expressing high levels of CD45RC do not. CD45RC has been revealed as a critical marker to distinguish within both CD4^+^^20^ and CD8^+^ T cells^18^ the pro-inflammatory cells (CD45RC^high^) from pro-regulatory cells (CD45RC^low/^^−^). Based on these observations, since more than 15 years, our team isolates CD8^+^ Tregs based on the following phenotype: CD3^+^CD56^−^CD8^+^CD45RC^low/-^ since systematic evaluation in fresh CD8^+^CD45RC^low/-^ Tregs of alternative or additional proposed markers by flow cytometry, including CD28, CD122, and else, was proven unsuccessful to enrich the tolerogenic activity.^14^ We have demonstrated the suppressive function of CD8^+^CD45RC^low/-^ cells freshly isolated and/or expanded for up to 21 days *ex vivo* and their potential to prevent transplantation rejection, graft versus host disease (GvHD) and multiple sclerosis.^5^^,^^13^^,^^14^^,^^15^^,^^21^^,^^22^^,^^23^^,^^24^ We demonstrated that expanded CD8^+^CD45RC^low/-^ Tregs show an increased suppressive activity compared to fresh one and differences in their phenotype, notably the overexpression of FOXP3.^14^ According to phenotype analysis by flow cytometry, CD8^+^CD45RC^low/-^ Tregs is a heterogeneous population based on surface markers in peripheral blood, and this heterogeneity is decreasing with polyclonal *ex vivo* expansion to reach to some extent a homogenous and clinically applicable population.

To investigate whether a fraction of the circulating CD8^+^CD45RC^low/-^ population is naturally tolerogenic, emerging from a thymic differentiation and is preferentially expanded, or if the induction of tolerance is peripheral and due to a combination of cells forming an “immunological niche” and allowing for the generation of induced CD8^+^CD45RC^low/-^ Tregs, a deep characterization of fresh CD8^+^ T cells is needed.

To delineate their heterogeneity at steady state and to clearly define specific markers of CD8^+^ Tregs, here we employed single cell RNA-seq coupled to CITEseq including VDJ sequencing on CD8^+^ T cell human peripheral blood. With this approach we could nail down two subsets of Tregs with different transcriptomic profile, which allowed us to gain unprecedented knowledge of those key immunological players. We identified surface markers predicting higher Treg activity and validated two of them in functional assays. Altogether our study will greatly improve our ability to understand the role of CD8^+^ Tregs in immunological response.

## Results

### Integrated single-cell RNA sequencing and CITE-seq reveals comprehensive subset distribution of CD8^+^ T cells in healthy donors

**Figure 1:**
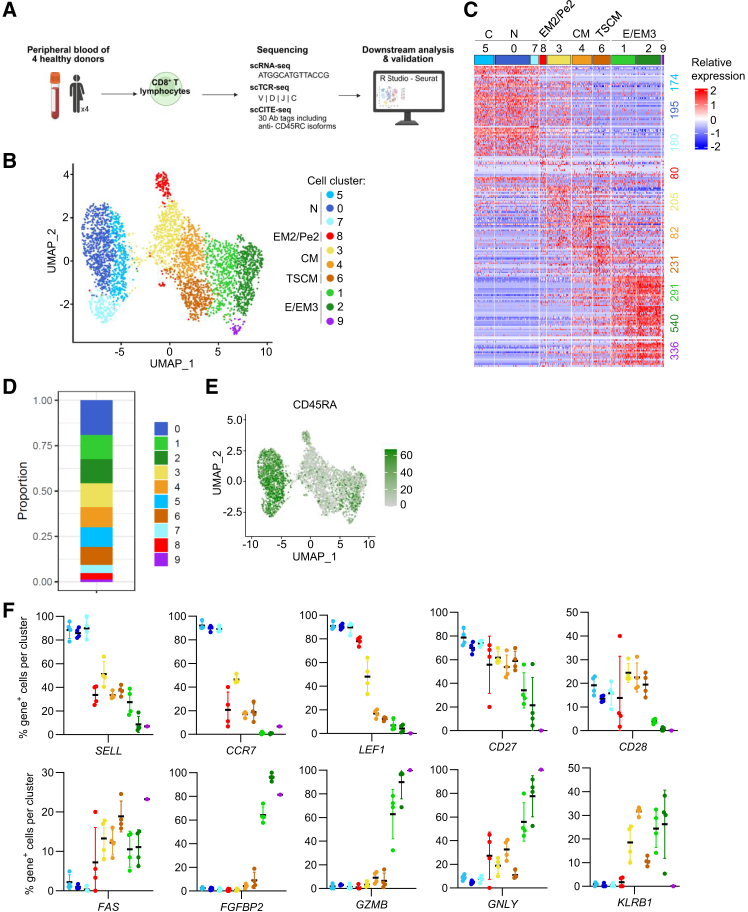
Single cell RNA-seq coupled to CITE-seq reveals CD8^+^ T cell subpopulations (A) Schematic representation of 5′ single-cell RNA-sequencing workflow (illustrated with Biorender). (B) Identification of CD8^+^ T cell subpopulations by clustering based on gene expression using uniform manifold approximation and projection (UMAP) (resolution = 1). Ten distinct clusters were defined identifying naive (N), central memory (CM), effector memory (EM) types 1 (EM1), 2 (EM2), 3 (EM3), and Pe2 (TEMRA) CD8^+^ T cells. Each dot corresponds to a single cell. (C) Heatmap showing the expression of genes defining cell cluster’s identities. Columns represents individual cells grouped by clusters and rows correspond to specific genes. Expression levels were scaled per gene with blue color indicating lower expression and red indicating higher expression. (D) Proportion of cells per cluster among CD8^+^ T cells. Data represent concatenated single-cell data from all four donors. (E) Distribution of CD45RA protein marker across clusters using UMAP. (F) Frequency of gene expression across CD8^+^ T cells clusters. Each dot corresponds to an individual donor. Each color corresponds to a specific cluster. Mean +/− standard deviation (SD).

To mitigate individual bias, we utilized barcoded antibodies (HTOs) to label and pool freshly isolated CD8^+^ T cells (CD4^−^ CD3^+^) from the blood of 4 healthy volunteers (Figure S1A). The cells were analyzed by single-cell RNA-seq coupled with 30 CITE-seq antibodies including several Tregs related-markers (Figure 1A). CD8^+^ T cell purity was confirmed at both transcriptomic and proteomic levels (Figure S1B). A total of 7517 single cells, evenly distributed among healthy individuals meeting the inclusion criteria (Figures S1C and S1D), were integrated in Seurat, log normalized and visualized by uniform manifold approximation and projection (UMAP) (Figure 1B). We confirmed that there was no significant bias (number of reads and mitochondrial genes content) and all reads passed quality check (see mat & met and Figure S1D). Initial clustering analysis identified 10 distinct cell clusters (Figures 1B and 1C) comprising 1%–19% of CD8^+^ T cells per cluster and between 44 and 763 cells per cluster (Figure 1D). Differential expression analysis revealed 2314 genes with significant expression differences between clusters, with more than 80 and up to 540 genes differentially expressed per cluster (Figure 1C).

Expression of key genes (*SELL*, *CCR7*, *CD27*, and *LEF1*) and the surface marker CD45RA identified naive T cells in clusters 0, 5, and 7 (Figures 1E and 1F). Using established T cell surface markers and developmental gene profiles, we further categorized clusters 1, 2, and 9 as effector/effector memory (E/EM3) T cells, characterized by the high expression of *FGFBP2*, *GZMB*, *GNLY*, *KLRB1*, low expression of *CD27*, and absence of expression of *CCR7*, displaying a CD45RA^low/^^−^CD28^low/-^ phenotype. Clusters 3, 4, and 6 were identified as central memory (CM) T cells, marked by a positive expression of *CCR7* and *CD27*, with a CD45RA^−^CD28^+^ phenotype. Cluster 8 was defined as the effector memory 2/precursor EM2 (EM2/pE2) stage, distinguished by *CD27* expression, with a CD45RA^low/^^−^CD28^−^ phenotype. Cluster 6, which is characterized by the high expression of CD95 (*FAS*), may also correspond to stem cell-like memory T cells (TSCM).

### Characterization of two CD8^+^ T cell subsets exhibiting distinct regulatory signatures

**Figure 2:**
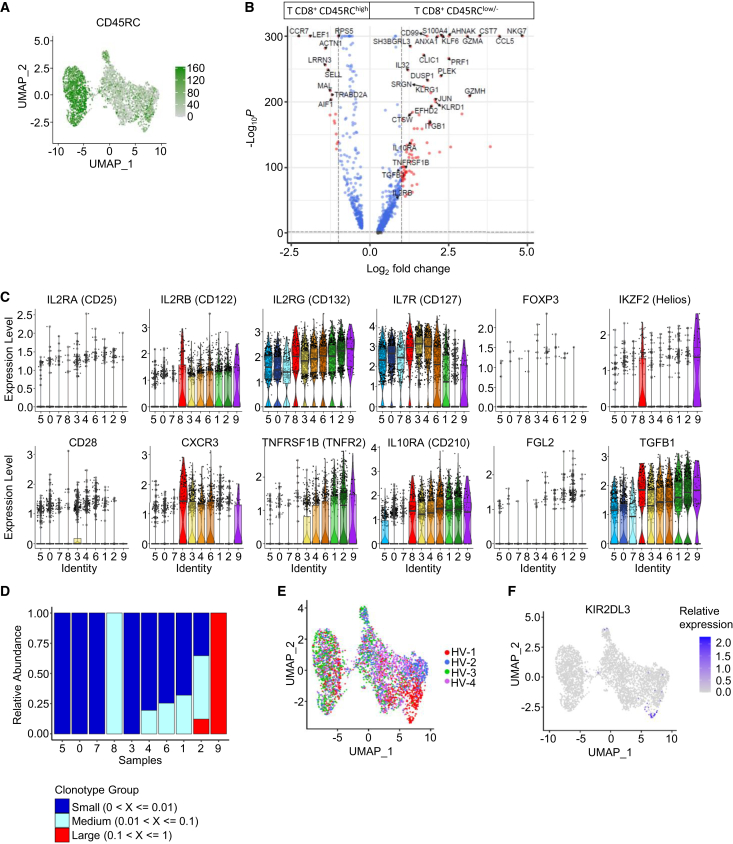
Characterization of CD8^+^ regulatory T cell clusters (A) Distribution of CD45RC protein marker across clusters using UMAP. (B) Volcano plot (min.pct = 0.25, logfc.threshold = 0.2) comparing gene expression between CD45RC^high^ (cluster 0, 5 and 7) vs. CD45RC^low/-^ (clusters 1–4, 6, 8, 9) CD8^+^ T cells. Thresholds were set to *p* value adjusted <0.05 and fold change >1.5. Red dots indicate genes meeting both criteria and blue dot indicates genes with significant adjusted *p* values. Some differentially expressed genes are labeled. (C) Violin plots displaying the expression of regulatory T cell-associated genes across the clusters. Violin plots depict gene expression distributions across the clusters, with each color corresponding to a specific cell cluster and each dot representing a single cell. The central line indicates the median, the box the interquartile range (IQR), and the whiskers extend to the minimum and maximum values. (D) Clonotype distribution across clusters with red indicating clonotypes found in 10%–100% of cells within a cluster, light blue in 1%–10% and dark blue in 0%–1%. (E) UMAP showing the affiliation of the CD8^+^ T cells to individual healthy volunteers. Each dot represents a single cell, and colors indicate the corresponding healthy volunteer, highlighting the distribution and affiliation of cells across the dataset. (F) Feature plot showing *KIR2DL3* gene expression in CD8^+^ T cells. Gene expression is represented on a color scale from gray (low expression) to blue (high expression) with each dot corresponding to a single cell.

We further analyzed the expression levels across the identified clusters, categorizing cells as CD45RC^high^, CD45RC^low^, or CD45RC^−^ based on CITE-seq antibody expression (Figures 2A and S2). CD45RC expression clearly distinguished clusters 0, 5, and 7 which displayed high CD45RC expression from the remaining clusters exhibiting low or negative expression (Figure S2A). The proportion of cells expressing high, low, or negative levels of CD45RC was evenly distributed among donors (Figure S2B).

Differential gene expression analysis revealed that CD8^+^CD45RC^low/-^ cells shared similar transcriptomic profiles, while CD8^+^CD45RC^high^ T cells were transcriptionally distinct (Figure S2C). Since we and others previously demonstrated that CD8^+^CD45RC^high^ T cells lack suppressive activity,^14^^,^^15^^,^^21^^,^^24^ these findings are consistent with pro-regulatory CD8^+^ T cells enriched within clusters 1–4, 6, and 8–9.

Further analysis focusing on the CD45RC marker identified 1179 genes differentially expressed between CD8^+^CD45RC^high^ (clusters 0, 5, and 7) and CD8^+^CD45RC^low/-^ T cells (Figure 2B), distributed evenly across healthy individuals. Volcano plot analysis comparing CD8^+^CD45RC^high^ and CD8^+^CD45RC^low/-^ T cells highlighted distinct gene signatures. CD8^+^CD45RC^high^ T cells showed significantly higher expression of membrane proteins, such as *MAL*, *LRRN3*, and *CCR7* or transcription factors such as *LEF1* which is critical for repressing CD4^+^ lineage-associated genes including *CD4*, *FOXP3*, and *RORC* in CD8^+^ T cells.^25^ In contrast, CD8^+^CD45RC^low/-^ T cells exhibited significantly higher expression of genes encoding membrane proteins such as *CD99*, *KLRG1*, *PLEK*, *KLRD1* (CD94), *IL10RA*, *IL2RB*, *ITGB1*, and *TNFRSF1B* (TNFR2), along with secreted proteins like *CCL5*, *CST7*, *GZMA*, and *TGFB1*.

Refining the analysis by focusing on specific cell clusters highlighted 3 clusters of particular interest, namely clusters 2, 8, and 9. Clusters 8 and 9, although small in size with 146 and 44 cells, respectively, showed high expression of *IKZF2* (HELIOS). In contrast, cell cluster 2 did not express *IKZF2* but showed a high expression of *TNFRSF1B* (TNFR2) and a lower expression of *IL7R* (CD127) and *CD28* (Figure 2C). We also observed expression of *FGL2* in cell cluster 2, a gene associated with CD4^+^ Treg suppressive function and which we have previously reported to be involved in CD8^+^ Treg suppressive function.^26^ Expression of *FOXP3* in contrast was not differentially expressed between cell clusters and was rarely detected (Figure 2C), consistent with prior studies demonstrating the low expression of FOXP3 in freshly isolated unstimulated CD8^+^ T cells.^14^

The analysis of TCR clonotypes relative abundance across cell cluster, based on the frequency distribution of small (<1%), medium (1%–10%) and large (>10%) clonotypes, revealed that most cell clusters had small clonotype frequencies, characteristic of polyclonal populations (Figure 2D). However, clusters 2, 8, and 9 displayed a higher frequency of expanded clonotypes, suggesting that these clusters are dominated by specific expanded clonotypes compared to the other clusters, (Table S1). Notably, cluster 9 exhibited a particularly large clonotype abundance, driven by a unique TCR repertoire from a single donor among the 4 healthy individuals. The clonotypic expansion observed in cluster 9 reflects an immune state of that specific donor, rather than a population commonly found in steady state conditions (Figure 2E). Cluster 9 also uniquely expressed *KIR2DL3*, which altogether with *IKZF2* expression, a profile previously reported in human CD8^+^ Tregs involved in anti-viral immune responses.^6^ Given that this cluster was not representative of all individuals but of one donor and potentially involved in suppression of pathogenic T cells in infectious diseases, we opted not to further investigate it in this study, as its characterization would exceed the statistical power if the current dataset.

The clustering results of the single cell RNA-seq data support the presence of transcriptionally distinct human CD8^+^ Treg subsets at steady state. Specifically, clusters 2 and 8 appear to represent these subsets, potentially exhibiting distinct maturation states and functions. We also excluded the possibility that mature and functional CD8^+^ Treg subsets exists within the remaining cell clusters.

### HELIOS and CD8 coexpression defines a distinct effector memory CD8^+^ Treg subset

**Figure 3:**
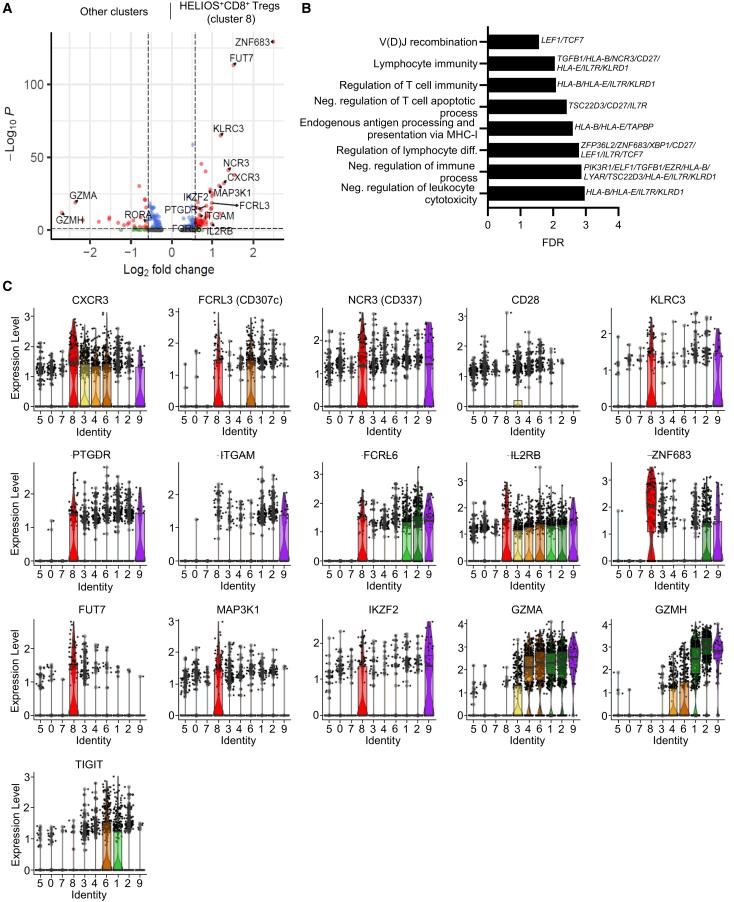
Identification of markers specific to HELIOS^+^CD8^+^ T regulatory cells (A) Volcano plot showing differentially expressed genes between cell cluster 8 vs. all other clusters. The thresholds have been set at adjusted *p* value <0.05 and fold change >1.5. Red dots indicate genes that exceeded both thresholds, while blue dots correspond to genes with a significant adjusted *p* value but not fold change. (B) Pathway analysis of differentially expressed genes in HELIOS^+^CD8^+^ Tregs compared to other clusters. Pathways are ranked by significance using an adjusted *p* value threshold of <0.05. Bars represent the degree of enrichment, with the x axis showing the fold enrichment and the y axis listing the biological processes or pathways. The key genes involved in each pathway are labeled on the right of the bar. (C) Violin plots displaying the expression of in HELIOS^+^CD8^+^ Tregs-associated genes across the clusters. Violin plots depict gene expression distributions across the clusters, with each color corresponding to a specific cell cluster and each dot representing a single cell. The central line indicates the median, the box the IQR, and the whiskers extend to the minimum and maximum values.

To further investigate the specific features of cell cluster 8 (within the CD8^+^CD45RC^low/-^ T cell pool), we compared by volcano plot the transcriptomes of cell cluster 8 with the other cell clusters, highlighting specific gene expression patterns (Figure 3A). As previously mentioned, clusters 8 exhibited a distinct expression of *IKZF2*, a transcription factor linked to the regulatory function of both CD4^+^ and CD8^+^ Tregs.^7^ Additionally, we observed high expression of *ZNF683*, a transcription regulator of tissue resident T cell and the notable expression of *FUT7*, a gene mediating synthesis of CD15s, a molecule known to be expressed by eTreg and associated with the identification of highly suppressive Treg subsets.^27^ *MAP3K1*, an important regulator of T cell proliferation and expansion appeared also to be specific of cluster 8.

Pathway analysis of differentially expressed genes between HELIOS^+^CD8^+^ Tregs (cluster 8) and other clusters demonstrated enrichment in pathways related to negative regulation of immune system processes, T cell apoptotic processes, leukocyte-mediated cytotoxicity, regulation of T cell and lymphocyte-mediated immunity, lymphocyte differentiation, antigen processing, and VDJ recombination (Figure 3B).

Among other markers characteristic of cell cluster 8 (Figure 3C), we identified *KLRC3* and *NCR3*, both associated with NK cells and previously linked to KIR^+^CD8^+^ Treg cells.^6^ These markers play roles in regulating humoral and cellular immunity. Additionally, *PTGDR*, a receptor for prostaglandin D2 and other prostaglandins with a lower affinity but with common anti-inflammatory activities, is involved in TGFβ-dependent immune responses and Treg differentiation.^28^^,^^29^ The analysis also revealed differential expression of *FCRL3* and *FCRL6*, two Fc-receptor like glycoprotein. FCRL3 is expressed on Tregs from fetal thymus origin and serves as a marker of effector Th17-like Tregs.^30^ Together with TIGIT and HELIOS, it has been associated with memory Tregs.^31^ FCRL6, known for its regulatory and inhibitory functions, is expressed by mature effector lymphocytes and can be engaged by LAG3.^32^

Further analysis of expression of cytokine/chemokine receptor revealed that cluster 8 exhibited higher levels of *IL2RB* (the IL-2 receptor) and *CXCR3*, which binds chemokines CXCL9, CXCL10, and CXCL11, influencing proliferation, maintenance, and suppressive activity of effector Treg cell.^33^ In contrast, we observed no expression of GZMA and GZMH, associated with a cytotoxic function.

In summary, cell cluster 8, characterized by high expression of *HELIOS* and specific cytokine/chemokine receptors, exhibits distinct regulatory features and is associated with pathways crucial for immune system modulation, effector Treg functionality, and T cell differentiation.

### Coexpression of *TNFRSF1B* and *ITGB1* defines a subset of induced CD8^+^ Treg

**Figure 4:**
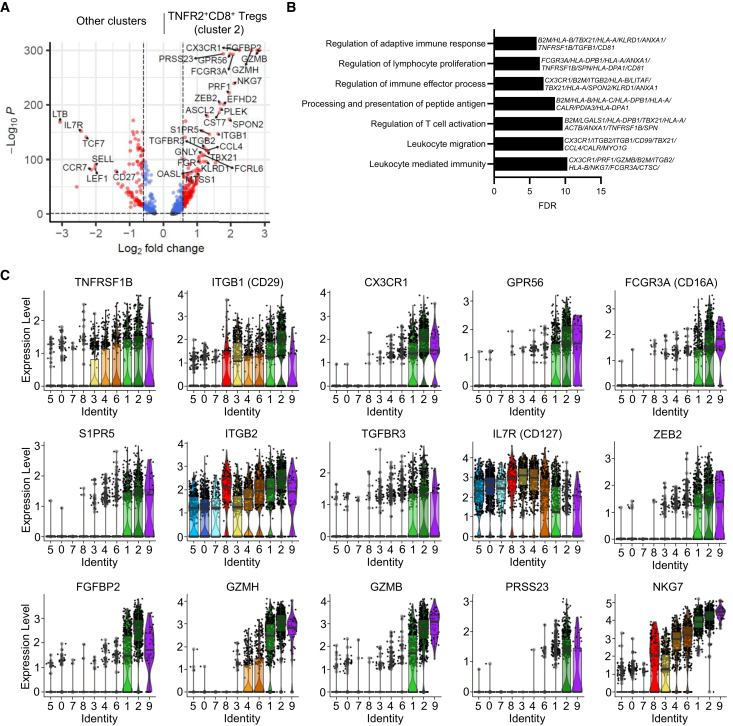
Identification of markers specific to TNFR2^+^CD8^+^ T regulatory cells (A) Volcano plot showing differentially expressed genes between cell cluster 2 vs. all other clusters. The thresholds have been set at adjusted *p* value <0.05 and fold change >1.5. Red dots indicate genes that exceeded both thresholds, while blue dots correspond to genes with a significant adjusted *p* value but not fold change. (B) Pathway analysis of differentially expressed genes in TNFR2^+^CD8^+^ Tregs compared to other clusters. Pathways are ranked by significance using an adjusted *p* value threshold of <0.05. Bars represent the degree of enrichment, with the x axis showing the fold enrichment and the y axis listing the biological processes or pathways. The key genes involved in each pathway are labeled on the right of the bar. (C) Violin plots displaying the expression of in TNFR2^+^CD8^+^ Tregs-associated genes across the clusters. Violin plots depict gene expression distributions across the clusters, with each color corresponding to a specific cell cluster and each dot representing a single cell. The central line indicates the median, the box the IQR, and the whiskers extend to the minimum and maximum values.

We next focused on cluster 2 and differential gene expression analysis of this cell cluster versus the other clusters revealed a distinctive differentiated, activated and induced profile with markers, such as *FGFBP2*, *CX3CR1*, and *FCGR3A,* commonly associated to effector function^34^ (Figure 4A). Cell cluster 2 showed reduced *IL7R*, *SELL*, *CD27*, and *TCF7*, consistent with a more differentiated and potentially effector-like phenotype. Key transcription factors like *ASCL2*, *ZEB2*, or *TBX21*, linked to activated T cell and polarized Tregs, were also preferentially expressed.^35^ Pathway analysis comparing differentially expressed genes between TNFR2^+^CD8^+^ Tregs (cluster 2) to the other clusters highlighted significant enrichment for pathways associated with regulation of T cell activation, lymphocyte proliferation, immune effector process, adaptive immune response, leukocyte migration and immunity, and antigen presentation (Figure 4B).

Notably, the genes previously associated with Treg function and tolerance such as *ITGB1*^36^ and *GPR56*^37^ were detected; with *ITGB2* also overexpressed, suggesting a potential involvement in the generation of induced Tregs^38^^,^^39^ (Figure 4C). Further examination of cytokine/chemokine receptor expression revealed elevated levels of *TGFBR3*, *S1PR5*, and *TNFRSF1B* (TNFR2).^40^ Unexpectedly, we also observed expression of cytotoxic genes, such as *GZMH*, *GZMB*, *PRF1*, known to be important for CD4^+^ Tregs functions in inflammatory environments^41^ but never observed before on CD8^+^CD45RC^low/-^ Tregs.^14^ Additionally, CD16A, a marker associated with NK cell was expressed.

Overall, our findings suggest that the signature of cluster 2 indicates an adaptive and activated Treg population that may employ distinct mechanisms of suppression or exhibit unique suppressive capacities. Together the analysis of clusters 2 and 8 supports the existence of several distinct CD8^+^ Treg subsets in humans, each with specific transcriptomic profiles, paralleling the diversity observed in CD4^+^ Treg cells.

### Reduced TCR repertoire diversity and differential chain usage in TNFR2^+^CD8^+^ Tregs compared to HELIOS^+^CD8^+^ Tregs

**Figure 5:**
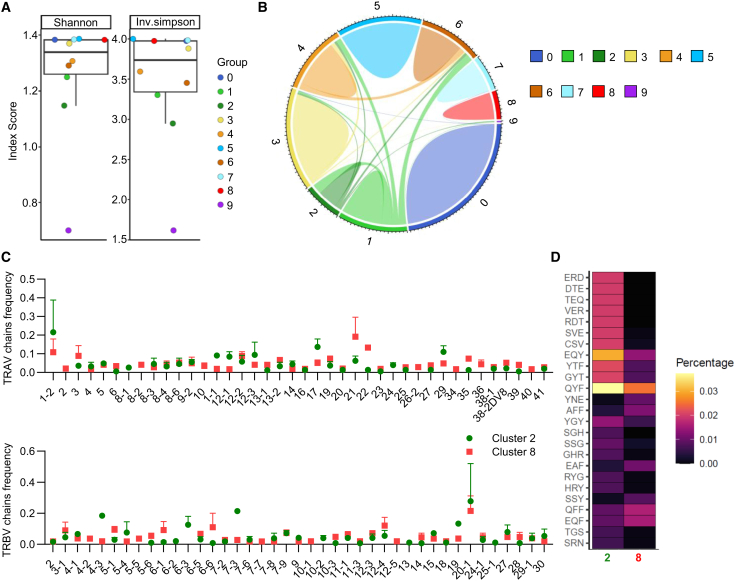
TCR diversity of CD8^+^ regulatory T cell clusters (A) Shannon diversity index (left) and Inverse Simpson index (right) scores for each CD8^+^ T cell cluster, representing TCR clonotype diversity. Each dot corresponds to a single cluster, and error bars represent the mean ± SEM for all groups. The Shannon index reflects the evenness and richness of TCR clonotypes within each cluster, with higher values indicating greater diversity. The inverse Simpson index measures the probability that two randomly selected clonotypes belong to different clonotypes, with higher values indicating less dominance by a single or few clonotypes. (B) Chord plot showing the extent of TCR clonotypes sharing between cell clusters for all healthy volunteers. Each colored segment represents a distinct cell cluster, and the connecting bands indicate shared TCR clonotypes between clusters. The thickness of the bands corresponds to the number of shared clonotypes, with thicker bands representing higher levels of clonotype overlap. (C) Relative frequency of variable domain of the alpha (TRAV, upper) and beta (TRBV, lower) TCR chains in cluster 2 and 8. Results are expressed as mean ± SEM (*n* = 4). (D) Kmer analysis of the CDR3 region identifies enriched motifs within TCR sequences across cluster 2 and 8. The heatmap displays the frequency of specific amino acid kmer motifs in cluster 2 and 8. The relative frequency of each kmer is represented by color intensity, with lighter shades indicating higher enrichment.

Given that natural Tregs, particularly CD4^+^ Tregs, selected in the thymus have been shown to express TCRs distinct from those of effector T cells, we further investigated the TCR alpha and beta chain usage among different cell clusters and sharing in between them. For this, we assessed the TCR diversity (Figure 5A) and performed a chord plot analysis of the TCR clonotypes (both alpha and beta chains) (Figures 5B and S3A). Evaluation of the evenness of the clone size distribution across clonotypes using the Shannon or Simpson’s diversity indexes (Figure 5A) demonstrated a significantly reduced diversity of TCR clonotypes for TNFR2^+^CD8^+^ Tregs (cluster 2) and cluster 9 (supporting an anti-viral immune response in a single individual), whereas HELIOS^+^CD8^+^ Tregs (cell cluster 8) or other cell clusters did not show similar reductions. Chord plot analysis revealed notable clonotypes sharing between cell clusters, particularly within the TNFR2^+^CD8^+^ Tregs (cell cluster 2) (Figure 5B). Further analysis of alpha and beta chain usage showed a significant bias toward TRAV1-2 and TRBV20-1 in TNFR2^+^CD8^+^ Tregs and TRAV21 and TRBV20-1 in HELIOS^+^CD8^+^ Tregs (Figure 5C). Additionally, analysis of CDR3α and CDR3β amino acid (aa) length, did not reveal any distinct length bias between TNFR2^+^CD45RC^low/-^ CD8^+^ T cells vs. TNFR2^−^CD45RC^low/-^ CD8^+^ T cells, while for HELIOS^+^CD8^+^ Tregs we observed an increased usage of 14 aa for the CDR3α chain, but no bias for the CD3β chain vs. HELIOS^−^CD8^+^ Tregs (Figure S3B). To further elucidate the TCR repertoire characteristics of TNFR2^+^CD8^+^ Tregs and HELIOS^+^CD8^+^ Tregs, we performed a K-mer analysis on the CDR3 sequence to identify and quantify recurring motifs. By using a sliding window approach across the amino acid sequences at fixed intervals, we identified several enriched motifs in TNFR2^+^CD8^+^ Tregs (cluster 2) including ERD, DTE, TEQ, VER, RDT, SVE, CSV, EQY, YTF, GYT, and QYF and to a lesser extent in HELIOS^+^CD8^+^ Tregs (cluster 8) including EQY, QYF, QFF, and EQF (Figure 5D). Our analysis revealed that TNFR2^+^CD8^+^ Tregs exhibited a distinct k-mer profile compared to HELIOS^+^CD8^+^ Tregs, underscoring the observed bias in alpha and beta chain usage and aligning with our previous findings of reduced TCR diversity. The absence of notable k-mer length biases in CDR3α and CDR3β sequences further supports the lack of preferential sequence lengths between the two Treg subsets. This k-mer analysis complements our findings and reinforces the idea of specialized TCR repertoires within these distinct CD8^+^ Treg populations. The k-mers represent highly conserved or functionally important subsequences within the CDR3 loops that may contribute to antigen binding and TCR engagement. Thus, identifying these motifs as enriched within cluster 2 supports the hypothesis of a biased TCR repertoire in this subset, potentially favoring specific antigen recognition or regulatory function.

### TNFR2 contributes to the functional activity of TNFR2^+^CD29^low^CD8^+^ highly suppressive Tregs

**Figure 6:**
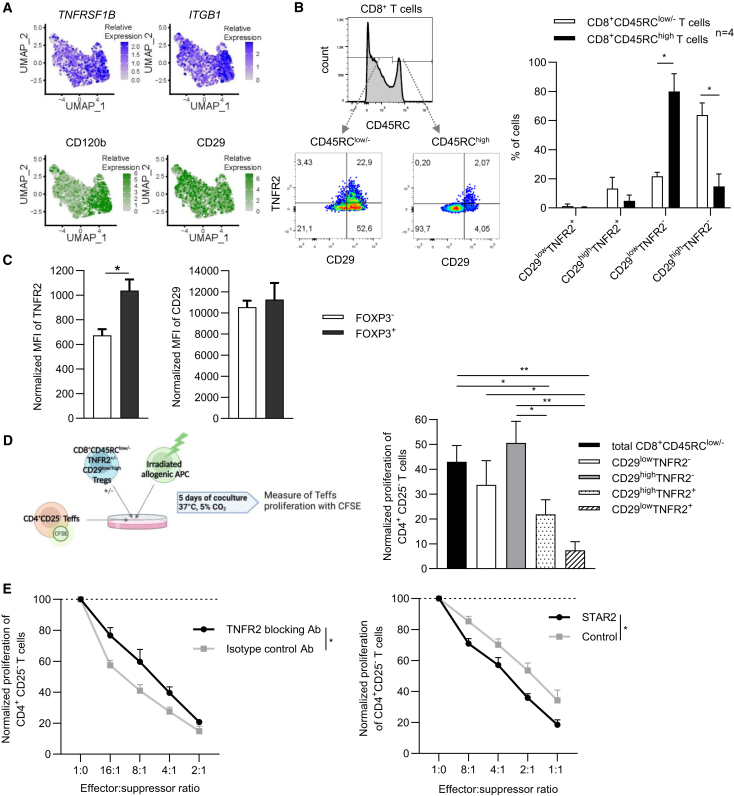
TNFR2^+^CD29^low^CD8^+^CD45RC^low/-^ Tregs exhibit enhanced suppressive function highlighting TNFR2’s role (A) UMAP projection of CD8^+^CD45RC^low/-^ T cells showing gene and protein expression of *TNFRSF1B* (CD120b) and *ITGB1* (CD29). Gene expression is scaled from gray to blue for genes, while protein expression is scaled from gray to green. Each dot corresponds to a single cell. (B) Left: Flow cytometry gating strategy to identify CD45RC^low/-^ and CD45RC^high^ CD8^+^ T cells and their subsets based on TNFR2 and CD29 surface expression. Right: Frequency of the four subsets (TNFR2^+^CD29^low^, TNFR2^+^CD29^high^, TNFR2^−^CD29^low^, TNFR2^+^CD29^high^) in CD45RC^low/-^ and CD45RC^high^ CD8^+^ T cells. Results are expressed as mean +/− SD. Mann-Whitney two-tailed test. ∗, *p* < 0.05. (C) Normalized MFI of TNFR2 and CD29 in FOXP3^+^ and FOXP3^-^ CD45RC^low/^^−^CD8^+^ T cell subsets, relative to isotype control. Results are expressed as mean +/− SEM. Mann-Whitney two-tailed test. ∗, *p* < 0.05. (D) Left: suppressive assay workflow. Responder T cells (CD4^+^CD25^−^ T cells) were labeled with CFSE and co-cultured in presence of irradiated allogenic Antigen Presenting Cells (APCs) with or without subsets of CD8^+^ Tregs for 5 days at 37°C with 5% of CO_2_. Proliferation was measured on CFSE dilution. Right: bar plot showing the percentage of proliferation of responder T cells with different subsets of CD8^+^ Tregs (ratio Teff:Treg 1:1) normalized to proliferation in absence of Tregs. Results are expressed as mean ± SEM (*n* = 6). Mann-Whitney two-tailed test. ∗, *p* < 0.05; ∗∗, *p* < 0.01. (E) Left: percentage of proliferation of responder T cells co-cultured with CD8^+^CD45RC^low/-^TNFR2^+^ T cells in the presence of saturating 10 μg/mL blocking anti-TNFR2 or isotype control Ab (Left) or in the presence of TNC-scTNF(143N/145R) STAR2 (Right), in a range of Teff:Treg ratios, normalized to proliferation without Tregs. Two-way row matched Anova test. ∗*p* < 0.05. Results are shown as mean ± SEM (n = 4–5).

To evaluate the suppressive potential of TNFR2^+^CD8^+^ Tregs, we focused on genes encoding membrane proteins highlighted in our single cell transcriptomic analysis. Specifically, we selected *ITGB1* (CD29) and *TNFRSF1B* (TNFR2) as markers (Figure 6A). At the proteomic level, surface expression of TNFR2 and CD29 distinguished four subsets of peripheral blood CD8^+^CD45RC^low/-^ T cells (Figure 6B). Notably, we identified a significantly higher proportion of CD29^high^TNFR2^−^ cells and fewer CD29^low^TNFR2- cells in the CD8^+^CD45RC^low/-^ T population compared to CD8^+^CD45RC^high^ T cells (Figure 6B). Although *FOXP3* was not exclusively expressed by cell cluster 2 in the single cell RNA-seq data, we further analyzed its protein level correlation based on prior literature for CD4^+^ Tregs.^42^ Our analysis revealed a positive correlation between FOXP3 with TNFR2 expression but not with CD29 (Figure 6C). To explore functional relevance, we sorted the four TNFR2/CD29 subsets from peripheral blood of healthy subjects and assessed their suppressive capacity on allogenic CD4^+^ effector T cells stimulated with irradiated allogeneic antigen-presenting cells (APC). Interestingly, both CD29^high^TNFR2^+^ and CD29^low^TNFR2^+^ CD8^+^CD45RC^low/-^ T cell subsets displayed significantly enhanced suppressive function than the total CD8^+^CD45RC^low/-^ T cell population (Figures 6D and S4C), suggesting a critical role for TNFR2 in the development or function of CD8^+^ Treg. We also observed a notable trend for stronger suppression by the TNFR2^+^CD29^low^ subset compared to the TNFR2^+^CD29^high^ cells.

To further investigate TNFR2’s role as a functional marker, and in light of its described functional relevance for CD4^+^ Tregs,^43^ we tested the effect of a blocking anti-TNFR2 antibody on the suppressive activity of TNFR2^+^CD8^+^CD45RC^low/-^ Tregs (Figure 6E). The addition of the blocking anti-TNFR2 antibody significantly decreased the suppressive activity of TNFR2^+^CD8^+^CD45RC^low/-^ Tregs, indicating that TNFR2 signaling is important for their suppressive function. However, as shown previously,^14^ other mechanisms likely contribute to the suppressive function, as blocking TNFR2 signaling did not fully restore conventional T cells proliferation.

We then stimulated TNFR2 with the TNFR2-specific agonist TNC-scTNF(143N/145R), a nonavalent TNFR2 agonist composed of the tenascin-C trimerization domain genetically fused to a single-chain encoded cassette comprising three human TNF protomers with mutations preventing TNFR1 binding (STAR2)^44^ (Figure 6F). Addition of STAR2 in the suppressive assay led to a significant increase in the suppressive activity of total CD8^+^CD45RC^low/-^ T cells on conventional CD4^+^CD25^−^ T cells proliferation, confirming the functional importance of TNFR2 in the regulation of CD8^+^ Treg activity.

### Confirmation of CD8^+^ Treg transcriptional and TCR signatures in an independent dataset

**Figure 7:**
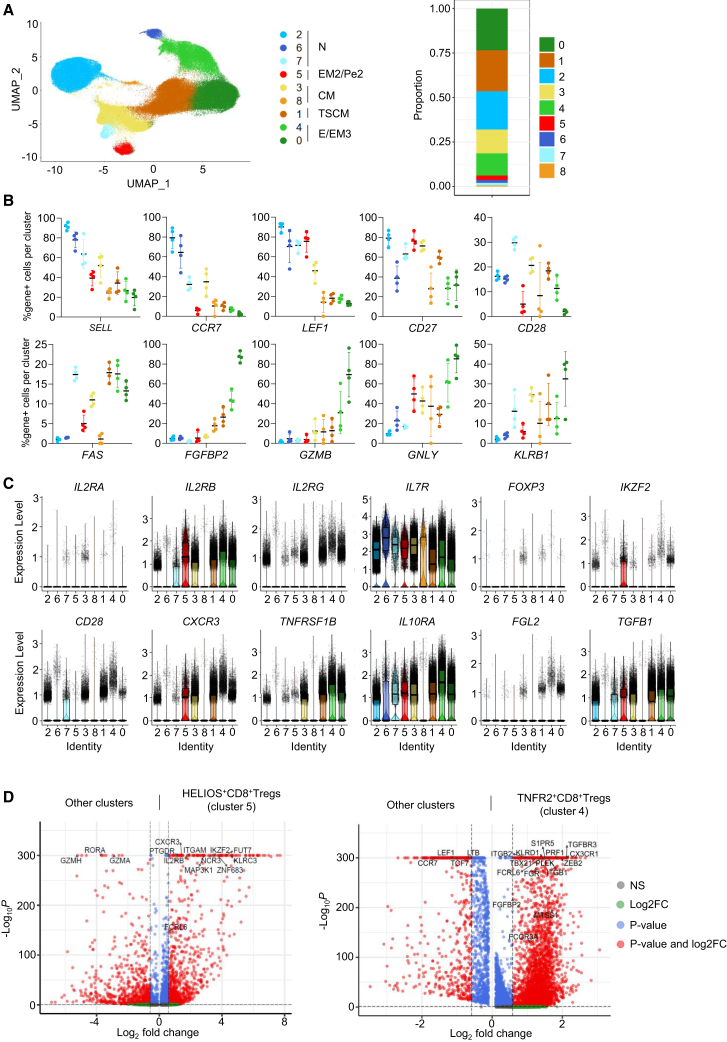
Identification of transcriptionally distinct CD8^+^ Treg subsets in an independent scRNA-seq dataset (A) Left, UMAP plot showing clustering of CD8^+^ T cells from a public dataset (10× Genomics) comprising ∼60,000 cells per individual and 4 individuals. Nine transcriptionally distinct clusters were identified. Right: proportion of cells per cluster among CD8^+^ T cells. Data represent concatenated single-cell data from all four donors. (B) Frequency of cells positive for the expression of canonical genes used to define naive (*SELL*, *CCR7*, *CD27*, *LEF1*), effector (*FGFBP2*, *GZMB*, *GNLY*, *KLRB1*), and memory (*CCR7*, *CD27*, *FAS*) T cell subsets. Each dot represents an individual donor. Results are expressed as mean +/- SD. (C) Violin plots displaying gene expression levels of selected markers across clusters. Colors correspond to cell clusters, and each dot represents a single cell. The central line indicates the median, the box the IQR, and the whiskers extend to the minimum and maximum values. (D) Volcano plots showing the transcriptional profiles of cluster 4 (TNFR2^+^) and cluster 5 (HELIOS^+^).

We extended our analysis by integrating a publicly available scRNA-seq dataset of peripheral blood CD8^+^ T cells from healthy donors from 10× genomics, including approximately 60,000 cells per individual. Using the same markers and clustering approach as in our initial analysis, we identified 9 distinct cell clusters (Figure 7A), comprising between 1% and 23% of CD8^+^ T cells and 913 to 36611 cells per cluster. Expression of key developmental genes identified naive T cells in clusters 2, 6, and 7 (*SELL*, *CCR7*, *CD27*, and *LEF1*); clusters 4 and 0 as E/EM3 T cells (*FGFBP2*, *GZMB*, *GNLY*, and *KLRB1)*; clusters 3 and 8 as CM T cells (*CCR7* and *CD27*), cluster 1 as TSCM (*FAS*) and cluster 5 as EM2/pE2 T cells (*CD27*^+^CD28^−^*CCR7*^*−*^) (Figure 7B).

We then further refined the analysis by focusing on specific genes of particular interest based on our initial analysis and confirmed the presence of subsets with transcriptional profiles similar to the HELIOS^+^ and TNFR2^+^ CD8^+^ Tregs described in our study in clusters 5 and 4, respectively (Figure 7C). As shown in Figure 7D, expression of *IKZF2* (HELIOS), *ZNF683* and *FUT7* recapitulates the signature of the *HELIOS*^+^ Treg subset (cluster 5), while a distinct cluster characterized by *TNFRSF1B*, *ITGB1*, *ITGB2*, *TGFBR3*, *S1PR5*, *FGFBP2*, *CX3CR1*, *FCGR3A*, *ZEB2*, and *TBX21* matches the phenotype of the *TNFR2*^+^ Treg population (cluster 4), supporting the existence of at least two CD8^+^ Treg-like subsets at steady state, as we described. Consistently with our findings, expression of *FOXP3* was not differentially expressed between cell clusters and was rarely detected.

**Figure 8:**
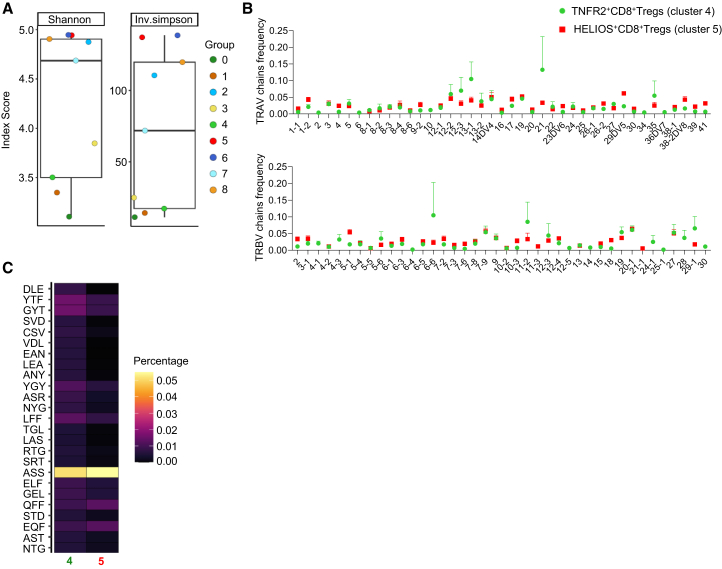
TCR repertoire analysis of CD8^+^ Treg subsets in an independent public dataset (A) Shannon diversity index (left) and inverse Simpson index (right) scores for each CD8^+^ T cell cluster from the public dataset, representing TCR clonotype diversity. Each dot corresponds to a single cluster, and error bars represent the mean ± SEM for all groups. (B) Relative frequency of variable domain usage for the alpha (TRAV, upper) and beta (TRBV, lower) TCR chains in TNFR2^+^ (cluster 4) and HELIOS^+^ (cluster 5) CD8^+^ Treg subsets. Results are expressed as mean ± SEM. (C) Heatmap displaying the frequency of specific amino acid k-mer motifs in CDR3 region in TNFR2^+^ (cluster 4) and HELIOS^+^ (cluster 5) CD8^+^ Treg subsets. The relative frequency is represented by color intensity.

Analysis of the paired TCRα and TCRβ sequences from the public dataset provided additional insights into the clonal architecture of the identified CD8^+^ Treg subsets (Figure 8). As in our original dataset, the TNFR2^+^CD8^+^ Treg subset (cluster 4) exhibited reduced TCR diversity and prominent clonal expansions, supporting a model of antigen-driven, peripherally induced Tregs (Figure 8A). V gene usage patterns showed partial overlap with our initial findings: while TRAV21 and TRAV13-1 were the most enriched α chains in the public dataset, TRBV20-1 originally identified in our dataset remained among the top four most used β chains in TNFR2^+^CD8^+^ Tregs and was the most frequent in HELIOS^+^CD8^+^ Tregs (cluster 5) (Figure 8B). This partial concordance suggests a degree of consistency in V gene usage, despite expected donor-to-donor variation and technical differences between datasets. The HELIOS^+^CD8^+^ Treg subset again displayed a polyclonal and diverse repertoire, with no dominant V gene usage pattern and overall higher diversity scores, reinforcing its likely thymic origin and homeostatic maintenance (Figures 8A and 8B). K-mer analysis of the CDR3 sequences revealed a shared enrichment for an ASS motif in both TNFR2^+^ and HELIOS^+^ CD8^+^ Treg subsets, suggesting some convergent structural features that may be functionally relevant, despite broader repertoire differences (Figure 8C).

Together, these analyses validate key aspects of our original findings in an independent cohort and provide additional support for the existence of clonotypically and transcriptionally distinct CD8^+^ Treg subsets in human peripheral blood.

## Discussion

In transplantation and inflammatory pathologies, one present major challenge is to develop strategies to control inflammation with more specificity and less toxicity compared to immunosuppressive standard treatments. Recent clinical trials using CD4^+^ Tregs as cell therapy demonstrated absence of toxicity and hints of efficacy.^45^ CD8^+^ Tregs present at least equivalent suppressive activity compared to their CD4^+^ counterparts.^5^^,^^12^^,^^14^^,^^21^^,^^46^ Notably in transplantation, CD8^+^ Tregs can take advantages of direct antigen presentation by MHC-I graft cells over time, as well as from donor APCs. In contrast, CD4^+^ Tregs can only become activated through direct presentation by donor APCs and transient endothelial cells MHC-II expression. Expanded CD8^+^ Tregs (CD3^+^CD56^−^CD8^+^CD45RC^low/-^ Tregs) will be used as cellular therapy for solid organ transplantation in the eight-Treg clinical trial within the reshape consortium (http.www.reshape.org). Efforts are ongoing to improve the therapeutic properties of CD8^+^ Tregs, including the use of CARs^21^ and genetic cell modifications to improve persistence and stability.^47^^,^^48^ These efforts benefit from deeper characterization and a better understanding of CD8^+^ Tregs that is made possible through single-cell transcriptomic and advanced bioinformatic tools. In addition, identifying key markers of CD8^+^ Tregs is critical for immune monitoring of patients after transplant or with autoimmune diseases, to guide therapeutic interventions, and predict the efficacy of CD8^+^ Treg therapy in the upcoming phase I.^47^

To the best of our knowledge, here we report the first single cell RNA-seq dataset focused on human CD8^+^ Tregs. Our analysis highlights the transcriptional heterogeneity of CD8^+^ T lymphocytes from peripheral blood with the confirmation of the existence of 2 major subsets: CD8^+^CD45RC^high^ and CD8^+^CD45RC^low/-^ T cells, consistent with prior work by our group and others.^16^^,^^17^^,^^18^^,^^20^ CD45RC emerged as a reliable marker of pro-inflammatory vs. pro-regulatory populations.^13^^,^^16^^,^^17^^,^^18^^,^^19^^,^^49^

While several markers have been proposed to identify CD8^+^ Tregs,^10^^,^^12^^,^^50^ only CD45RC showed clear cluster specificity. While markers such as TNFR2, CD28, CD122, and CD25 showed broader or overlapping expression profiles, other markers such as FOXP3 was rarely detected at the mRNA level consistent with previous observations.^14^ These findings suggest that CD8^+^ Tregs are not a homogeneous population and that several regulatory subsets may coexist, explaining the historical lack of consensus regarding their phenotypic definition. This delineation of pro-inflammatory vs. pro-regulatory populations is critical for the generation of a therapeutic strategy as illustrated by our work with anti-CD45RC monoclonal antibodies in preclinical models of organ transplantation,^16^ acute GVHD,^49^ and APECED.^17^

Compared to our previous 3′DGE-seq transcriptomic characterization of CD8^+^CD45RC^low/-^ T cells in human (before vs. after *ex vivo* expansion) and rat (before vs. after anti-CD45RC mAb treatment),^14^^,^^16^ the dataset generated here provides higher resolution and single-cell level insights, enabling the identification of additional transcriptional regulators and surface proteins, It has been clearly established that CD8^+^ T cells with regulatory properties are within the CD8^+^CD45RC^low/-^ T cells fraction.^13^^,^^14^^,^^16^ We excluded cells with more innate properties such as NKT cells (excluded based on CD56^+^CD16^+^ protein expression by CITEseq) and MAIT cells (based on CD161^+^ cells and TRAV1-2^+^TRAJ33^+^ expression), to further refine the analysis of conventional CD8^+^ Treg subsets, while preserving the possibility of detecting CD56^+^ Tregs^51^^,^^52^ or CD161^+^ Tregs,^53^ although future studies could revisit these cell populations.

Interestingly, this work highlighted three transcriptionally distinct CD8^+^CD45RC^low/-^ T cells clusters. In particular, in cell cluster 2, we observed higher levels of several genes previously associated with Tregs, such as TNFR2 *(TNFRSF1B)*, PD-1 *(PDCD1)*, *FASLG*, *IFNG*, and *FGL2*, along with lower levels of genes associated with T effector cells (*CD28*, *CD27*, and *CD127*), supporting a differentiated Treg phenotype. We selected two genes encoding for membrane proteins with previous occurrence of association with Tregs, TNFR2^42^^,^^54^^,^^55^^,^^56^ and CD29^57^ to further specifically isolate this cell cluster for functional studies.

We demonstrated that CD8^+^CD45RC^low/^^−^TNFR2^+^CD29^low^ T cell represent a highly suppressive subset capable of mediating 90% suppression *in vitro* at 1:1 ratio, even at steady state (without pre-stimulation). This surpasses the suppressive capacity of other previously described markers like GITR which required overnight polyclonal stimulation to achieve a similar 80% suppression at a similar ratio Teff:Treg.^14^ The association between suppressive function with TNFR2 expression is of great interest since several agonists targeting TNFR2 are under clinical development for CD4^+^ Treg and may also enhance CD8^+^ Treg functional properties.^58^^,^^59^ Blocking TNFR2 signaling partially reversed suppression mediated by CD8^+^ Tregs, suggesting it contributes to, but is not solely responsible for regulatory function. This is in line with previous work from our group showing that cytokines such as IFN-γ and IL-10 were also involved in CD8^+^ Treg-mediated suppression.^13^^,^^14^^,^^22^^,^^23^^,^^26^ Our team demonstrated that expression of FGL-2, IFN-γ, and IL-10 is important for CD8^+^CD45RC^low/-^ Treg-mediated suppression. To understand the role of TNFR2 for CD8^+^ Treg function, we also performed experiments using a TNFR2 agonist. Although the observed effect was modest (around 20%), it aligned with the level of expression of TNFR2 within the total CD8^+^CD45RC^low/-^ T cell population.

We also observed that the TNFR2^+^ subset is CD28^−^, consistent with previous work describing CD28 loss as a feature of activated or terminally differentiated Tregs.^8^ Although IL-10 mRNA is not detectable in this dataset, *IFNG* was upregulated in the relevant cluster, in agreement with our earlier functional data.^13^^,^^14^

*FOXP3* mRNA was rarely detected in our dataset, but we observed that FOXP3 protein was enriched in CD8^+^CD45RC^low/^^−^TNFR2^+^ cells compared TNFR2^-^ cells, suggesting transcriptionally low but functionally relevant expression. HELIOS (*IKZF2*) was in contrast clearly detected in cell clusters 8 and 9. Cluster 9 showed similarity to a recently described CD8^+^ KIR2DL3^+^ Treg subset involved in the control of anti-viral responses.^6^ While intriguing, the cluster’s restriction to a single donor precluded deeper analysis here.

To support the robustness of our findings, we incorporated an independent public dataset of around 60,000 CD8^+^ T cells per donor. This external dataset confirmed the presence of transcriptionally distinct TNFR2^+^ and HELIOS^+^ subsets with comparable marker expression profiles. Despite some variation in V gene usage (i.e., TRAV21 in the public cohort vs. TRAV1-2 in ours), TRBV20-1 was enriched across both datasets, particularly in HELIOS^+^ cells. The public dataset also confirmed reduced TCR diversity in TNFR2^+^ cells and a polyclonal profile in HELIOS^+^ cells, supporting the distinction between these subsets.

Although CD8^+^CD45RC^low/^^−^TNFR2^+^CD29^low^ cells represent a small population that could be difficult to isolate and amplify for cell therapy, they could serve as biomarkers for immune monitoring or target for *in vivo* modulation. Their capacity to recognize HLA class I molecules broadly expressed on graft tissues may offer an advantage in transplantation by enabling donor-specific, direct allorecognition.^15^^,^^24^^,^^60^ TNFR2 agonists are under development^58^^,^^59^ and expand CD8^+^ Treg function in autoimmune diseases or in the context of transplantation, whereas TNFR2 may be beneficial in cancer or infectious diseases.

CD29 (*ITGB1*), although widely expressed, has been linked to attachment of cells to matrix and signal transduction, but its role in T cell function is not clear.^61^^,^^62^ It has been identified to correlates with cytotoxic potential both in CD8^+^ T cells^63^ and CD4^+^ T cells.^64^ Thus, its relevance and role of Tregs remains to be fully investigated. Finally, additional candidate markers identified in our dataset, such as CD126/CD130 (*IL6R*/*IL6ST*), may help define CD8^+^ Treg subsets with higher stability in inflamed environment. As inflammation-driven Treg instability remains a major challenge, defining how CD8^+^ Tregs respond to inflammatory cytokines, such as IL-6 or TNF-α will be critical.^65^

As has been shown for CD4^+^ Tregs, where studies on heterogeneity have revealed functionally distinct subsets^66^ with specialized role in different pathologic situations,^67^ it is likely that several populations of CD8^+^ Tregs exist with phenotypic properties and different role in different situations. Understanding this heterogeneity could guide the development of specific therapeutic approaches adapted to particular disease conditions. Therefore, profiling CD8^+^ T cells in patients suffering from AID or transplanted, and in patients suffering from cancer will be essential to evaluate the presence, expansion or disappearance of the CD8^+^ Treg subsets described here and to better understand their relevance in human pathology. In addition to functional capacity, the metabolic fitness of CD8^+^ Tregs may determine their persistence *in vivo.*^68^ Metabolic profiling of identified subsets, particularly TNFR2^+^ cells, could reveal pathways that support their suppressive phenotype under nutrient-limited or hypoxic conditions.

While our analysis focused on phenotypic and transcriptional markers, defining the antigen specificity of CD8^+^ Tregs remains a key goal for future translational applications.^15^^,^^24^ TCR sequencing revealed reduced diversity in TNFR2^+^ cells, but the cognate antigens remain unknown. Identifying them could allow selective *in vivo* expansion using peptide-based strategies.

To conclude, using scRNAseq and integrated bioinformatic analysis, we defined the heterogeneity of CD8^+^CD45RC^low/-^ Tregs and identified TNFR2 and CD29 as two surface markers that enable functional enrichment of a highly suppressive subset. These markers may serve as useful tools for monitoring or modulating CD8^+^ Treg activity in clinical application, particularly transplantation and autoimmune diseases.

### Limitations of the study

While this study provides the most comprehensive single-cell characterization of human CD8^+^ Tregs to date, several limitations should be acknowledged. First, the analyses were performed on peripheral blood CD8^+^ T cells from healthy individuals; the extent to which these findings apply to CD8^+^ Tregs present in tissues or under pathological conditions (i.e., autoimmunity, cancer, transplantation) remains to be determined. Second, the identification of CD8^+^ Treg subsets was based primarily on transcriptional and surface protein markers, and while functional assays confirmed suppressive activity for the TNFR2^+^ subset, the functional relevance of other subsets (i.e., HELIOS^+^) requires further experimental validation. Third, although the addition of an independent dataset helped validate the main transcriptional signatures and clonotypic features, technical differences between datasets and donor heterogeneity may limit the full comparability of the results. Finally, the study does not explore the stability or plasticity of these CD8^+^ Treg subsets over time or upon stimulation, which could be important for clinical applications.

## Resource availability

### Lead contact

Further information and requests for resources and reagents should be directed to and will be fulfilled by the lead contact, Carole Guillonneau (carole.guillonneau@univ-nantes.fr).

### Materials availability

All materials used are listed in the key resources table. The anti-human CD45RC antibody clone ABIS is a proprietary reagent licensed to a private company and is not commercially available.

### Data and code availability

• Raw and processed single-cell RNA-seq and TCR V(D)J data have been deposited in ArrayExpress: E-MTAB-15460.
• Public datasets used in this study are available from the 10× Genomics data repository (https://www.10xgenomics.com/resources/datasets).
• All scripts and code used for data analysis are available and listed in the key resources table.

## Acknowledgments

We thank Sonia Salle for blood sampling. This work was partially funded by the Labex IGO program supported by the National Research Agency via the investment of the future program ANR-11-LABX-0016-01. This work was supported by an “Etoiles Montantes” from Pays de la Loire to C.G. and the 10.13039/501100001659Deutsche Forschungsgemeinschaft (DFG, German Research Foundation) – project number 324392634 – TRR221 project B02 to H.W. This work was also realized in the context of the support provided by the Fondation Progreffe. This project has received funding from the European Union’s Horizon 2020 research and innovation program under grant agreement no 825392 “RESHAPE”, the 10.13039/501100001665French National Research Agency ANR-23-CE17-0062-02, the Fondation pour la Recherche Médicale Equipe EQU202303016266 and Prix Victor Erminia Mescle 202206015603.

## Author contributions

C.G. led and conceived the project, analyzed the data, wrote and revised the manuscript. C.S contributed to data collection, experimentation, analysis and writing of the manuscript. L.D. contributed to analysis and revision of the manuscript. S.B. contributed to conceptualization, analysis of the data and review of the manuscript. L.F., M.S., D.-J.W., S.S., F.H., P.D., G.M.G., K.L., and M.-F.M. contributed to data collection, experimentation and analysis. H.W. provided critical reagent. H.D.V., I.A., and L.D. contributed to conceptualization.

## Declaration of interests

The authors declare no competing interests.

## STAR★Methods

### Key resources table

**Table:** 

REAGENT or RESOURCE	SOURCE	IDENTIFIER
**Antibodies**		

Anti-CD3	BD Biosciences	Cat# 561416, SK7 clone; RRID: AB_10612021
Anti-CD4	BD Biosciences	Cat# 560768, RPA-T4 clone; RRID: AB_1937323
Anti-CD45RC	IqProducts and this paper	Cat# IQP-117F
Anti-CD25	BD Biosciences	Cat# 557753, M-A251clone; RRID: AB_396859
Anti-TNFR2	Miltenyi Biotec	Cat# REA52; RRID: AB_2904702
Anti-CD29	Miltenyi Biotec	Cat# REA1060; RRID: AB_2751454
Anti-human Hashtag antibodies TotalSeq-A	Biolegend	Custom mix
Isotype control IgG	Biolegend	RRID: AB_2733893
Anti-FOXP3	eBiosciences	Cat# 15549036 clone PCH101; RRID: AB_11042114
Anti-TNFR2 blocking	Biolegend	Cat# 358408; RRID: AB_2563224
TNFR2-specific agonist TNC-scTNF(143N/145R)	Provided by collaborator	As referenced in [Rauert et al.^44^]

**Biological samples**		

PBMC	Etablissement Francais du Sang	NA

**Chemicals, peptides, and recombinant proteins**		

Na-EDTA	Sigma Aldrich	Cat# E4884-500G
Ficoll-Paque	Eurobio, Courtaboeuf, France	Cat# CMSMSL01-01
4′,6-diamidino-2-phenylindole (DAPI)	Thermo Fisher	Cat# D1306
glutaMAX	Life Technologies	Cat# 35050
Non-Essential Amino Acids (NEAA)	Gibco	Cat# 11140050
sodium pyruvate	Gibco	Cat# 11360070
HEPES	Gibco	Cat# 15630080
Penicilin/Streptomycin	Gibco	Cat#15140-122
human AB serum	Sigma-Aldrich	Cat# H4522
Carboxyfluorescéine Diacétate Succinimidyl Ester	Thermo Fisher	Cat# C34554
FC block	BD Pharmigen	Cat# 564765/RRID: AB_28696126

**Critical commercial assays**		

LEGENDScreen™ Human PE Kit	Biolegend	cat # 700007
Nucleospin II RNA Kit	Macherey Nagel	Cat# 740955
Chromium Next GEM Single Cell 5′ Reagent Kits v2	10× Genomics	Cat# 1000263
Chromium Single Cell V(D)J Reagent Kit	10× Genomics	Cat# 1000072
FOXP3/Transcription Factor Staining Buffer Set	ThermoFisher scientific	Cat# 00-5523-00

**Deposited data**		

Raw data	ArrayExpress	https://www.ebi.ac.uk/biostudies/ArrayExpress/studies/E-MTAB-15460?key=d237fef3-ffbf-4827-afda-c14c7f04355e

**Software and algorithms**		

R	Bioconductor	V 4.0.3
Seurat	Satija Lab	Version 4.1.1 https://satijalab.org/seurat/
GraphPad Prism 7.0	phPad Software, Inc	http://www.graphpad.com
scRepertoire package	GitHub/CRAN	Version 1.7.2 https://github.com/ncborcherding/scRepertoire
FlowJo	FlowJo software	https://www.flowjo.com/
CellRanger package	10× Genomics	Version 6.1.2

### Experimental model and subject details

#### Human samples

Blood was collected at the Etablissement Français du Sang (Nantes, France). Heparinized blood samples were taken from healthy volunteers after signing an informed consent approved by the ethical committee of relevant institutions.

### Method details

#### Isolation of human peripheral blood mononuclear cells (PBMC) of healthy volunteers

Blood samples from healthy volunteers were collected in EthyleneDiamineTetraAcetic acid (EDTA) coated tubes for omic analyses or from buffy coats at Etablissement Francais du Sang (EFS) for suppression assays. PBMC were isolated with Ficoll-Paque density gradient centrifugation (Eurobio, Courtaboeuf, France). Red blood cells and platelets were removed with hypotonic lysis solution and centrifugation.

#### Staining and cell sorting of cells

PBMC were diluted in Phosphate Buffered Saline (PBS) – FCS 2% - EDTA 2 mM and labeled with surface antibodies anti-CD3 (BD Biosciences, SK7 clone), anti-CD4 (BD Biosciences, RPA-T4 clone), anti-CD45RC (IqProducts, MT2 clone) and with 4′,6-diamidino-2-phenylindole (DAPI). For suppression assay, anti-CD25 (BD Biosciences, M-A251clone) mAb was added. For intracellular staining, cells were permeabilized 30 min at 4°C with FOXP3/Transcription Factor Staining Buffer Set (ThermoFisher scientific), stained for 45 min at room temperature and then fixed with 2% PFA. Lymphocytes were selected according to their morphology in FSC-A and SSC-A, doublets cells were excluded and DAPI^+^ cells were removed. DAPI^−^CD3^+^CD4^−^ were sorted for CITE seq, DAPI^−^CD3^+^CD4^−^CD45RC^low/-^ T cells and DAPI^−^CD3^+^CD4^+^CD25^−^ T cells were sorted for suppression assay. In some cases, four subsets in CD8^+^CD45RC^low/-^ Tregs were labeled and sorted according to the expression of TNFR2^+/−^ (Miltenyi Biotec, REA520) and CD29^low/high^ (Miltenyi Biotec, REA1060). Purity was greater than 95%.

Cells were sorted with FACS ARIA II cell sorter (BD Biosciences) and data were analyzed with FLOWJO software (Tree Star, Inc., Ashland, OR, USA).

#### Single cell RNA-sequencing of blood CD8^+^ T cells

Freshly sorted CD8^+^ T lymphocytes of four healthy volunteers were labeled with anti-human Hashtag antibodies TotalSeq-C (Biolegend, San Diego, CA) and were pooled together. Cell viability was >90%. Cells were then incubated with a panel of 30 CITE-seq monoclonal antibodies, including both commercial and custom-labelled clones, for 30 min at 4°C. After staining, cells were washed thoroughly to remove unbound antibodies prior to single-cell encapsulation. The panel included antibody against CD45RC (ABIS clone, labeled in-house), CD120b (3G7A02 clone), HLADR (L243 clone), CD39 (A1 clone), CD357 (108-17 clone), CD279 (EH12.2H7 clone), CD152 (BNI3 clone), TIGIT (A15153G clone), CD223 (C9B7W clone), CD70 (113-16 clone), CD137 (4B4-1 clone), CD226 (11A8 clone), CD25 (BC96 clone), CD71 (CY1G4 clone), CD134 (Ber-ACT35 (ACT35) clone), CD11c (S-HCL-3 clone), CD16 (3G8 clone), CD56 (5.1H11 clone), CD28 (CD28.2 clone), CD45RA (HI100 clone), CD45RO (UCHL1 clone), CD103 (Ber-ACT8 clone), CD178 (NOK-1 clone), CD29 (TS2/16 clone), CD122 (TU27 clone), CD127 (A019D5 clone), CD126 (UV4 clone), CD130 (2E1B02 clone), CD21 (Bu32 clone) and CD197 (G043H7 clone). After staining, cells were immediately processed according to Chromium Next GEM Single Cell 5′ Reagent Kits v2 protocol (10× genomics, San Francisco, CA). Cell suspension were loaded onto chromium single cell chip K in 4 different wells (20 000 cells per well) and run immediately on the Chromium controller (10× genomics, San Francisco, CA). Three libraries were prepared: one for mRNA (RNA), one for barcoded antibodies (HTO and CITE-seq) and one for TCR (VDJ-seq). They were sequenced with NovaSeq 6000 (Illumina, San Diego, CA) according to 10× genomics recommendations.

#### Single cell RNA-sequencing data analysis

##### 1/primary analysis

FASTQ files, first generated from BaseCalling (BCL) files with CellRanger package (6.1.2), were demultiplexed and aligned to human reference genome (hg38). CITE-seq-Count function (1.3.4) was used to count antibody hashtag sequences.

##### 2/secondary analysis

Count matrixes were analyzed with Seurat R package (4.1.1). Cells with more than 10% of mitochondrial genes were excluded. Gene expression was log normalized and scaled. HTO expression was normalized and demultiplexed. Doublet cells and negative cells for HTO were removed. Downstream analysis was performed on 15718 single cells: 7535 fresh CD8^+^ T lymphocytes. On average 1454 genes were expressed per cell in fresh CD8^+^ T cells. A nonlinear dimensionality reduction Uniform Manifold Approximation and Projection (UMAP) and a clustering were performed to visualize distinct cell clusters with a resolution of 1 for total CD8^+^ T cells, 0.8 after MAIT cells exclusion, and 0.5 for CD8^+^CD45RC^low/-^ T cells only. Differentially expressed genes were calculated using “wilcox” method to characterize marker genes of each cell cluster implemented in the Seurat package’s “FindAllMarkers” or “FindMarkers” functions. CITE-seq antibodies gates were set up according to expression profiles detected by flow cytometry with negative and positive fractions and for some CITE-seq antibodies including anti-CD45RC with low and high fractions.

##### 3/TCR repertoire analysis

This dataset contained matched TCR for each single cell. For TCR analysis, scRepertoire package was used (1.7.2). TCR repertoire was analyzed by studying the length of the amino acid sequence of the CDR3 regions of TRA and TRB chains and by exploring clonotypes expansion. The interconnectivity between clusters regarding TCR sequences/clonotypes sequences were analyzed and visualized using chord plots. Finally, the iterations of each TCR sequence/clonotype in each cell cluster is presented based on the absolute frequency.

The accession number for sequencing raw data deposited in ArrayExpress is: E-MTAB-15460.

The public dataset used was generated by 10× Genomics and downloaded from their support site (https://support.10xgenomics.com/): CD8^+^ T cells of Healthy Donor 1, 2, 3 and 4, Single Cell Immune Profiling Dataset by Cell Ranger v3.0.2, 10× Genomics, (2019, May 9).

#### Suppressive assays

CD8^+^ Treg cell subsets sorted according to TNFR2 and CD29 expression were cultured in RPMI 1640 medium supplemented with glutaMAX, 1% NEAA, 1% sodium pyruvate, 1% HEPES 1% Penicilin/Streptomycin, and 5% human AB serum with autologous CD4^+^CD25^−^ conventional/responder T cells labeled with CFSE and irradiated allogenic antigenic presenting cells (APC). When indicated, blocking or anti-TNFR2 monoclonal antibody (358408, Biolegend) or its isotype control (400544, Biolegend) or the human TNFR2-specific agonist TNC-scTNF(143N/145R)^44^ were added. After 5 days of co-culture at 37°C, 5% CO_2_, CFSE fluorescence intensity was measured in CD3^+^CD4^+^DAPI^−^ T cells with BD FACS CANTO II (BD Biosciences) to measure the proliferation of CD4^+^CD25^−^ effector T cells with or without the presence of CD8^+^CD45RC^low/^^−^TNFR2^+^ Tregs or total CD8^+^CD45RC^low/-^ Tregs.

### Quantification and statistical analysis

Non-parametric Mann Whitney test was performed to compare TNFR2 and CD29 MFI in FOXP3^+^ and FOXP3^-^ CD8^+^CD45RC^low/-^ T cells. two-way ANOVA test was performed to compare TRAV and TRBV chain frequency in TNFR2^+^ and TNFR2^-^ CD8^+^CD45RC^low/-^ T cells. Volcano plot showing – log10 *p*-value and log2 fold change in y and x axis respectively. *p*-values were calculated with FindMarkers function of Seurat R package. Mann Whitney test, non-parametric, *p* value two tailed was performed to compare CD8^+^ Treg subsets suppressive functions at ratio Teff:Treg 1:1. Two-way row-matched ANOVA was performed to compare suppressive function of CD8^+^CD45RC^low/^^−^TNFR2^+^ T cells with blocking antibody anti-TNFR2 or isotype control and with STAR2 or isotype control in a range of cell ratio.
