# Supplementary material for: Two subsets of regulatory CD8+ T cells with differential transcriptome revealed by single cell analysis
Source: iScience. 2025 Sep 8;28(10):113512. doi: 10.1016/j.isci.2025.113512 (PMC12506525; doi:10.1016/j.isci.2025.113512)
Supplement: Document S1. Figures S1–S4 and Table S1 [file mmc1.pdf]

iScience, Volume *28*

Supplemental information

Two subsets of regulatory CD8$^+$ T cells with differential transcriptome revealed by single cell analysis

Céline Sérazin, Lisa Dugast, Léa Flippe, Mathias Streitz, Désirée-Jacqueline Wendering, Stephan Schlickeiser, Frederik Heinrich, Pawel Durek, Gabriela Maria Guerra, Katrin Lehmann, Mir-Farzin Mashreghi, Harald Wajant, Hans Dieter Volk, Ignacio Anegon, Laurent David, Séverine Bézie, and Carole Guillonneau

Supplementary Figure 1, Sérazin et al.

A

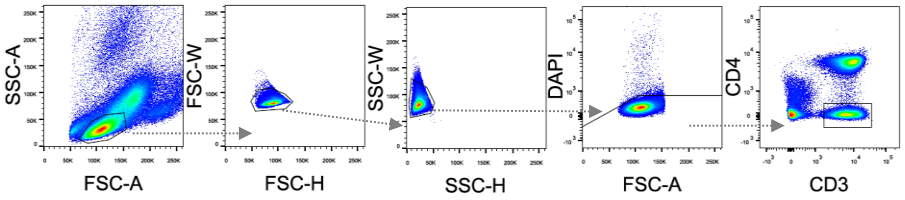

B

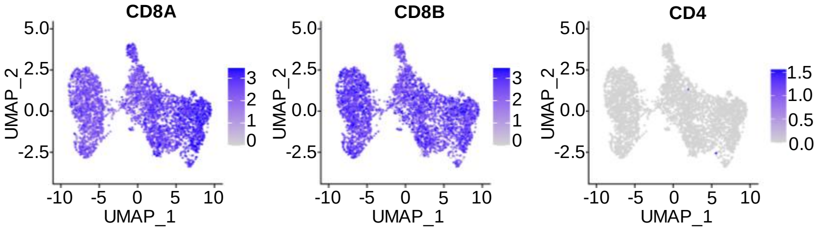

C

HV-1	HV-2	HV-3	HV-4	Total single cell
1908	1465	2000	2144	7517

D

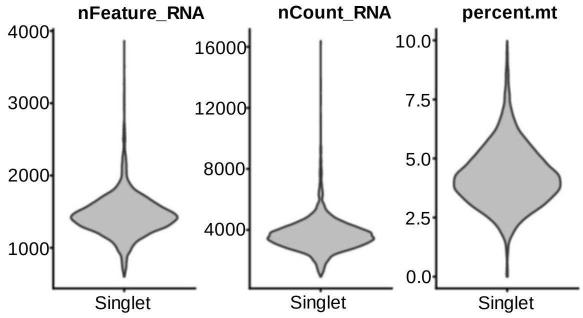

E

Cluster	#0	#1	#2	#3	#4	#5	#6	#7	#8	#9
Cell number	763	535	533	524	442	433	394	181	146	44
% of CD8 T cells	19	13	13	13	11	11	10	4,5	3,7	1,1

Suppl. Figure 1: CD8$^+$ T cell sorting, gene and protein Expression.

(A) CD8$^+$ T cell sorting strategy. CD8$^+$ T cells were defined by gating on lymphocytes morphology, FSC and SSC singlets, living cells, CD3$^+$CD4$^-$. **(B)** Feature plots showing gene expression of *CD8A*, *CD8B* and *CD4*. Gene expressions are scaled from grey to blue. Each dot corresponds to a single cell. **(C)** Number of single cells per healthy volunteer (HV). **(D)** Violin plots representing from left to right to the number of genes, the number of RNA molecules and the percentage of mitochondrial genes in each single cell for the all HV. **(E)** Number of single cells per cluster and percentage among CD8$^+$ T cells of each cluster.

Supplementary Figure 2, Sérazin et al.

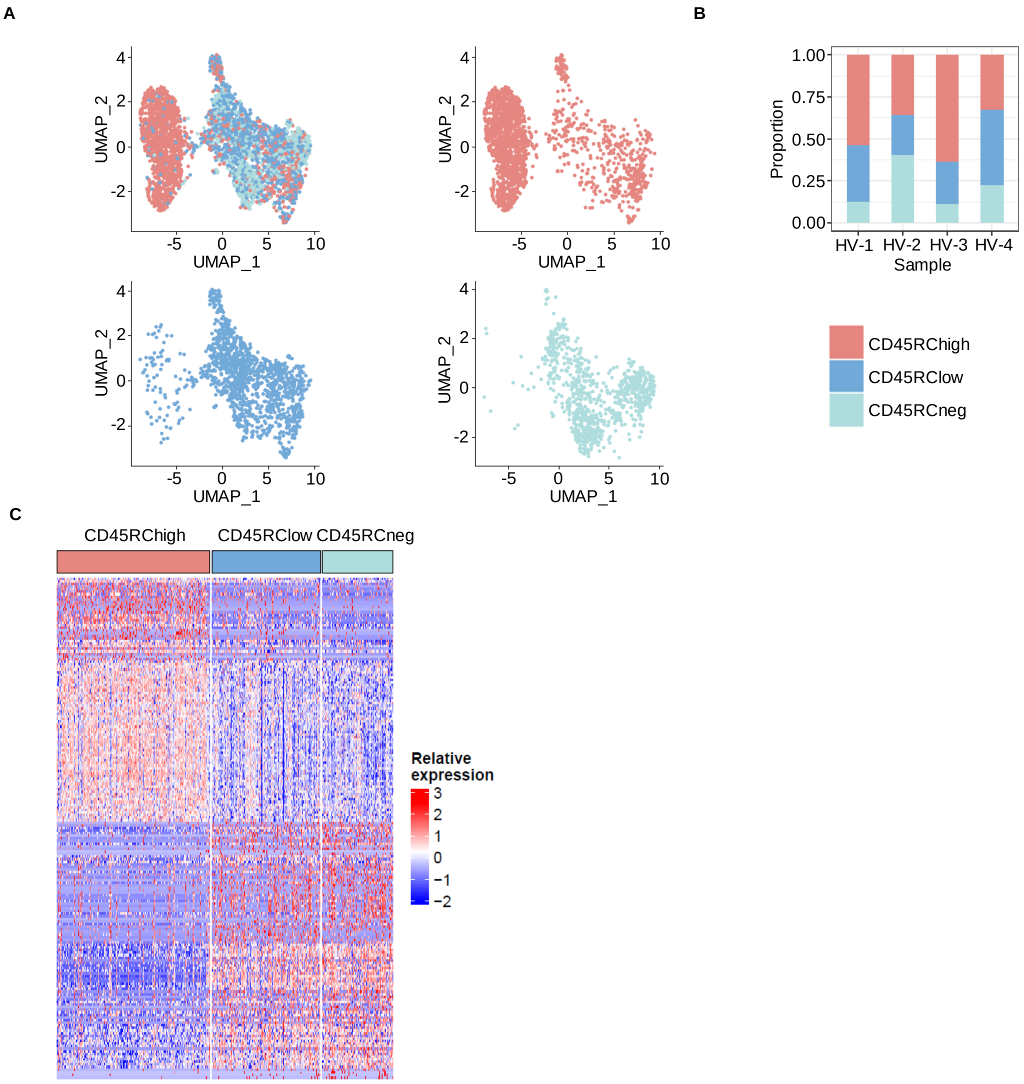

Suppl. Figure 2: Differential Gene Expression and Proportional Analysis of CD8$^+$ T Cell Subsets Based on CD45RC Expression.

(A) UMAP plots depicting CD45RC^{high} (orange), CD45RC^{low} (dark blue) and CD45RC^{neg} (light blue) cell subsets within total CD8$^+$ T cells using the anti-CD45RC CITE-seq mAb. Each dot corresponds to a single cell. **(B)** Proportion of CD45RC^{high} (orange), CD45RC^{low} (dark blue) and CD45RC^{neg} (light blue) cell subsets within total CD8$^+$ T cells in each healthy volunteer. **(C)** Heatmap of differentially expressed genes accross CD45RC^{high}, CD45RC^{low} and CD45RC^{neg} CD8$^+$ T cell subsets. Columns correspond to cell clusters and rows correspond to genes. Expression values were scaled per gene, blue indicating lower expression and red higher expression.

Supplementary Figure 3, Sérazin et al.

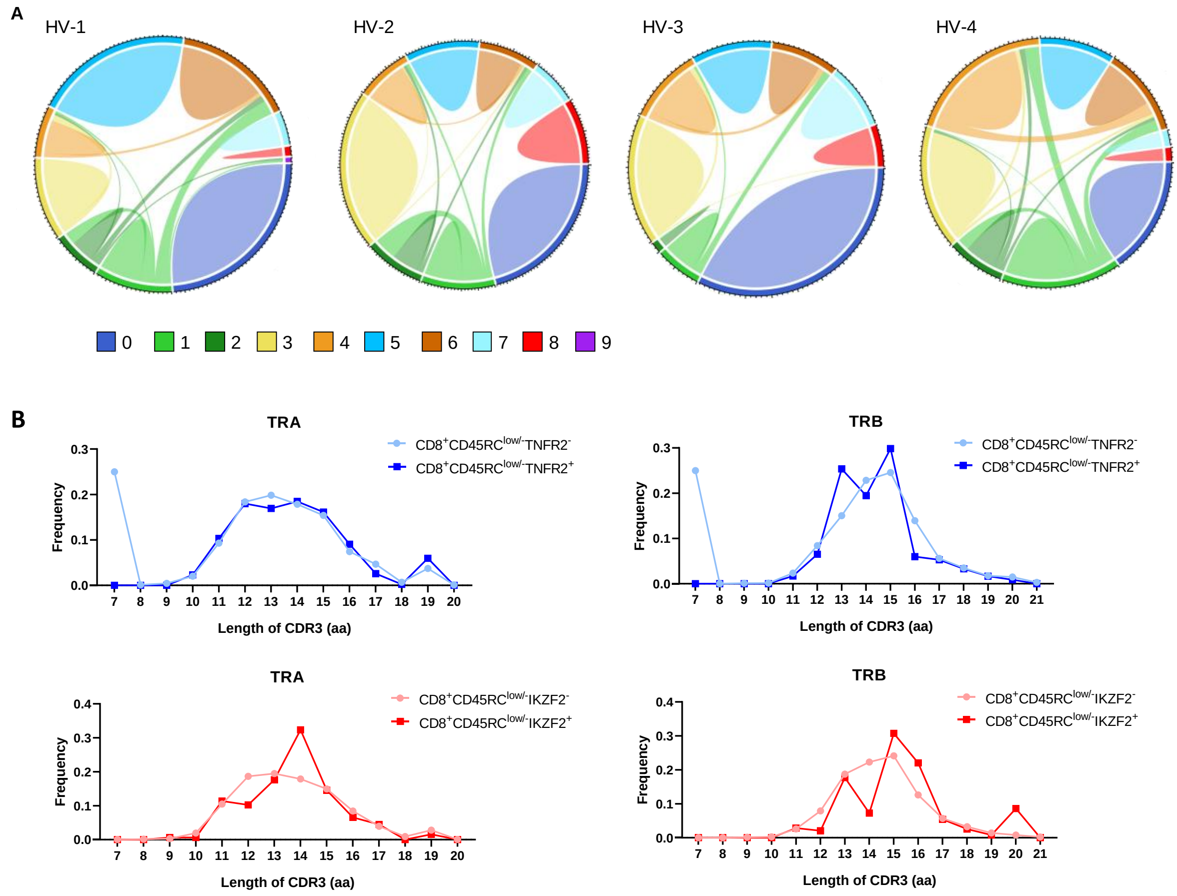

Suppl. Figure 3: TCR clonotype sharing and CDR3 length distribution across cell clusters. **(A)** Chord plot showing the extent of TCR clonotypes sharing between cell clusters for each healthy volunteer. Each coloured segment represents a distinct cell cluster, and the connecting bands indicate shared TCR clonotypes between clusters. The thickness of the bands corresponds to the number of shared clonotypes, with thicker bands representing higher levels of clonotype overlap. **(B)** CDR3α (TRA, left) and CDR3β (TRB, right) amino acid (aa) length frequency between cluster 2 or cluster 8 and all other clusters. TNFR2^{+}CD8^{+} Tregs are represented in dark blue and the other clusters in light blue.

Supplementary Figure 4, Sérazin et al.

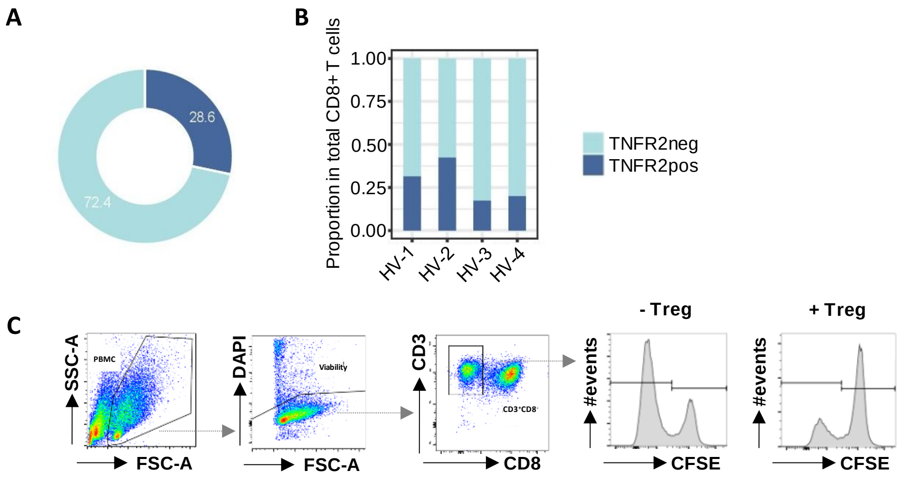

Suppl. Figure 4: Distribution and Proportions of TNFR2 Expression in CD45RC$^{low/-}$CD8^{+} T Cells.

(A) Percentage of TNFR2^{+} and TNFR2^{-} CD45RC$^{low/-}$CD8^{+} T cells represented as a donut chart. TNFR2^{+} are represented in dark blue and TNFR2^{-} are represented in light blue. **(B)** Proportion of TNFR2^{+} and TNFR2^{-} CD45RC$^{low/-}$CD8^{+} T cells in each healthy volunteer. TNFR2^{+} are represented in dark blue and TNFR2^{-} in light blue. **(C)** Representative histograms of CFSE profile in T CD4^{+} responder cell proliferation cultured in the presence or absence of CD8^{+} Tregs, after gating on morphology and living CD4^{+} T cells.

Suppl. Table 1: Most frequent CDR3α and β used sequences within clusters.

Cluster n°	CDR3β sequence	CDR3α sequence	Iterations	Shared with cluster n°
1	CATDANDYKLSF	CASSLGQGNQPQHF	3	
1	CILSQDTGNQFYF	CASSLGTGSGYEQYF	2	
2	CAASPNLEYSGGGADGLTF	CSARDLRDRDEKLFF	34	
2	CATGPYSGAGSYQLTF	CASSQDTGSRNTIYF	31	
2	CVVRRDDKIIF	CASSPSQAYGYTF	20	
2	CAVKDSYGKLTF	CASSLESSRGYTF	17	
2	CAAGLGNKLTF	CASSSGHRYGYTF	16	
3	CAVDSLANDYKLSF	CSARGPRGGEQGEEQYF	3	
3	CAASPGTYKYIF	CASSWGDSYNEQFF	2	
3	CAERINTGGFKTIF	CASSFRSSGRNTGELFF	2	
3	CAESSRSTLTF	CASSYSYEQYF	2	
3	CAFMEYGNKLVF	CASSQASSGQETQYF	2	
4	CAVKDSYGKLTF	CASSLESSRGYTF	15	
4	CAVRDGRDDKIIF	CASSPGGTEAFF	9	5 and 7
4	CAGAPGHNYGQNFVF	CASSYPTGYTF	8	
4	CVVRRDDKIIF	CASSPSQAYGYTF	8	
4	CAFHSDTGNQFYF	CASSPTSGRGETQYF	6	
5	CAPFYNFNKFYF	CASRSEAGNEKLFF	21	
5	CAASGDSGYSTLTF	CASSTSSRNQPQHF	10	
5	CAVSDSYGNNRLAF	CAIDGGPTGELFF	9	
5	CAVRDGRDDKIIF	CASSPGGTEAFF	6	4 and 7
5	CAVLGFGNVLHC	CASSLEETNEQFF	5	
6	CAVRDWDQAGTALIF	CASSQEGEGTGETQYF	2	
7	CAVRDGRDDKIIF	CASSPGGTEAFF	10	4 and 5
7	CAVRDWGSNTGFQKLVF	CASSSETVNGNQPQHF	9	
7	CATDRGGGNNNDMRF	CASSRRDARGKLFF	8	
7	CAVQGAETGGFKTIF	CASSSWGANTEAFF	6	
7	CAVIRAGGTSYGKLTF	CASSQGLYYEQYF	4	
9	CALSDPDNQGGKLIF	CSARVGGRQPQHF	2	
